# Multivariate genome-wide association analysis by iterative hard thresholding

**DOI:** 10.1093/bioinformatics/btad193

**Published:** 2023-04-17

**Authors:** Benjamin B Chu, Seyoon Ko, Jin J Zhou, Aubrey Jensen, Hua Zhou, Janet S Sinsheimer, Kenneth Lange

**Affiliations:** Department of Computational Medicine, David Geffen School of Medicine at UCLA, Los Angeles, CA 90095-1554, United States; Department of Computational Medicine, David Geffen School of Medicine at UCLA, Los Angeles, CA 90095-1554, United States; Department of Biostatistics, Fielding School of Public Health at UCLA, Los Angeles, CA 90095-1554, United States; Department of Biostatistics, Fielding School of Public Health at UCLA, Los Angeles, CA 90095-1554, United States; Department of Medicine, David Geffen School of Medicine at UCLA, Los Angeles, CA 90095-1554, United States; Department of Biostatistics, Fielding School of Public Health at UCLA, Los Angeles, CA 90095-1554, United States; Department of Computational Medicine, David Geffen School of Medicine at UCLA, Los Angeles, CA 90095-1554, United States; Department of Biostatistics, Fielding School of Public Health at UCLA, Los Angeles, CA 90095-1554, United States; Department of Computational Medicine, David Geffen School of Medicine at UCLA, Los Angeles, CA 90095-1554, United States; Department of Biostatistics, Fielding School of Public Health at UCLA, Los Angeles, CA 90095-1554, United States; Department of Human Genetics, David Geffen School of Medicine at UCLA, Los Angeles, CA 90095-1554, United States; Department of Computational Medicine, David Geffen School of Medicine at UCLA, Los Angeles, CA 90095-1554, United States; Department of Human Genetics, David Geffen School of Medicine at UCLA, Los Angeles, CA 90095-1554, United States; Department of Statistics at UCLA, Los Angeles, CA 90095-1554, United States

## Abstract

**Motivation:**

In a genome-wide association study, analyzing multiple correlated traits simultaneously is potentially superior to analyzing the traits one by one. Standard methods for multivariate genome-wide association study operate marker-by-marker and are computationally intensive.

**Results:**

We present a sparsity constrained regression algorithm for multivariate genome-wide association study based on iterative hard thresholding and implement it in a convenient Julia package MendelIHT.jl. In simulation studies with up to 100 quantitative traits, iterative hard thresholding exhibits similar true positive rates, smaller false positive rates, and faster execution times than GEMMA’s linear mixed models and mv-PLINK’s canonical correlation analysis. On UK Biobank data with 470 228 variants, MendelIHT completed a three-trait joint analysis (n=185 656) in 20 h and an 18-trait joint analysis (n=104 264) in 53 h with an 80 GB memory footprint. In short, MendelIHT enables geneticists to fit a single regression model that simultaneously considers the effect of all SNPs and dozens of traits.

**Availability and implementation:**

Software, documentation, and scripts to reproduce our results are available from https://github.com/OpenMendel/MendelIHT.jl.

## 1 Introduction

Current statistical methods for genome-wide association studies (GWAS) can be broadly categorized as single variant or multi-variant in their genomic predictors. Multi-variant sparse models ignore polygenic background and assume that only a small number of single-nucleotide polymorphisms (SNPs) are truly causal for a given trait. Model fitting is typically accomplished via regression with penalties, such as the least absolute shrinkage and selection operator (LASSO) (Wu et al. 2009, Zhou et al. 2010b, 2011, Alexander and Lange 2011, Qian et al. 2020), minimax concave penalty (Zhang 2010, Breheny and Huang 2011), iterative hard thresholding (IHT) (Keys et al. 2017, Chu et al. 2020), or Bayesian analogues (Guan and Stephens 2011). Linear mixed models (LMMs) dominate the single-variant space. LMMs control for polygenic background while focusing on the effect of a single SNP. LMMs are implemented in the contemporary programs GEMMA (Zhou and Stephens 2012), BOLT (Loh et al. 2018), GCTA (Yang et al. 2011, Jiang et al. 2019), and SAIGE (Zhou et al. 2018). The virtues of the various methods vary depending on the genetic architecture of a trait. No method is judged uniformly superior (Galesloot et al. 2014).

Although there is no consensus on the best modeling framework for single-trait GWAS, there is considerable support for analyzing multiple correlated traits jointly rather than separately (Galesloot et al. 2014, Porter and O’Reilly 2017, Turchin and Stephens 2019). When practical, joint analysis (i) incorporates extra information on cross-trait covariances, (ii) distinguishes between pleiotropic and independent SNPs, (iii) reduces the burden of multiple testing, and (iv) ultimately increases statistical power. Surprisingly, simulation studies suggest these advantages hold even if only one of multiple traits is associated with a SNP or if the correlation among traits is weak (Galesloot et al. 2014). These advantages motivate this article and our search for an efficient method for analyzing multivariate traits.

Existing methods for multivariate-trait GWAS build on the polygenic model or treat SNPs one by one. For instance, GEMMA (Zhou and Stephens 2014) implements multivariate linear mixed models (mvLMM), mv-PLINK (Ferreira and Purcell 2009) implements canonical correlation analysis (CCA), and MultiPhen (O’Reilly et al. 2012) and Scopa (Mägi et al. 2017) invert regression so that the genotypes at a single SNP become the trait and observed traits become predictors. Due to their single-variant nature, these methods cannot distinguish whether a SNP exhibits a true effect on the trait or a secondary association mediated by linkage disequilibrium (LD). As a result, many correlated SNPs near the causal one are also selected. This inflates the false positive (FP) rate unless one applies fine-mapping strategies (Spain and Barrett 2015) in downstream analysis to distill the true signal. Joint regression methods like IHT and LASSO are less susceptible to finding SNPs with only secondary association because all SNPs are considered simultaneously.

To our knowledge, there are no sparse regression methods for multivariate-trait GWAS. In this article, we extend IHT (Blumensath and Davies 2009) to the multivariate setting and implement it in the Julia (Bezanson et al. 2017) package MendelIHT.jl, part of the larger OpenMendel statistical genetics ecosystem (Zhou et al. 2020). We have previously demonstrated the virtues of IHT compared to LASSO regression, and single-SNP analysis for univariate GWAS (Keys et al. 2017, Chu et al. 2020). Since IHT assumes sparsity and focuses on mean effects, it is ill-suited to capture polygenic background as represented in classic variance components models. In the sequel, we first describe our generalization of IHT. Then, we study the performance of IHT on simulated traits given real genotypes. These simulations explore the impact of varying the sparsity level *k* and the number of traits *r*. To demonstrate the potential of IHT on real large-scale genomic data, we also apply it to three hypertension-related traits and 18 metabolomic traits from the UK Biobank. Our simulation experiments and real data studies showcase IHT’s speed, low FP rate, and scalability to large numbers of traits. Our concluding discussion summarizes our main findings, limitations of IHT, and questions worthy of future research.

## 2 Materials and methods

### 2.1 Model development

Consider multivariate linear regression with *r* quantitative traits and *p* predictors. Up to a constant, the loglikelihood L(B,Γ) for *n* independent subjects is


(1) $$L(B,\Gamma )=\frac{n}{2}\text{log}(\det\;\Gamma )-\frac{1}{2}\text{tr}[\Gamma (Y-BX){\left(Y-BX\right)}^{T}].$$

The loglikelihood L(B,Γ) is a function of the r×p regression coefficients matrix B and the r×r unstructured precision (inverse covariance) matrix Γ. In Equation (1), Y is the r×n matrix of traits (responses), and X is the p×n design matrix (genotypes plus non-genetic predictors). All predictors are treated as fixed effects.

IHT maximizes L(B,Γ) subject to the constraints that *k* or fewer entries of B are non-zero and that Γ is symmetric and positive definite. The unknown parameter *k* is chosen via cross-validation. Optimizing L(B,Γ) with respect to B for Γ fixed relies on three core ideas. The first is gradient ascent. Elementary calculus tells us that the gradient $\nabla_{B}L(B,\Gamma )$ is the direction of steepest ascent of L(B,Γ) at B for Γ fixed. IHT updates B in the steepest ascent direction by the formula $B_{m+1}=B_{m}+t_{m}\nabla_{B}L(B_{m},\Gamma_{m})$, where *m* is iteration number, $t_{m}>0$ is an optimally chosen step length, and $\left(B_{m},\Gamma_{m}\right)$ is the current value of the pair (B,Γ). The gradient is derived in the online supplementary material as the matrix


(2) $$\nabla_{B}L(B,\Gamma )\;\;=\Gamma (Y-BX)X^{T}.$$

The second core idea dictates how to choose the step length $t_{m}$. This is accomplished by expanding the function $t\mapsto L[B_{m}+t_{m}\nabla_{B}L(B_{m},\Gamma_{m})]$ in a second-order Taylor series around $\left(B_{m},\Gamma_{m}\right)$. In the online supplementary material, we show that the optimal $t_{m}$ for this quadratic approximant is where $C_{m}$ abbreviates the gradient $\nabla_{B}L(B_{m},\Gamma_{m})$. The third core idea of IHT involves projecting the steepest ascent update $B_{m+1}=B_{m}+t_{m}\nabla_{B}L(B_{m},\Gamma_{m})$ to the sparsity set $S_{k}=\{B:||B|{|}_{0}\leq k\}$. The projection operator $P_{S_{k}}(B)$ sets to zero all but the largest *k* entries in magnitude of B. This goal can be achieved efficiently by a partial sort on the vectorized version $\text{vec}(B_{m+1})$ of $B_{m+1}$. For all predictors to be treated symmetrically in projection, they should be standardized to have mean 0 and variance 1. Likewise, in cross-validation of *k* with mean square error prediction, it is a good idea to standardize all traits.


(3) $$\begin{array}{c} t_{m}=\frac{||C_{m}|{|}_{F}^{2}}{\text{tr}(X^{T}C_{m}^{T}\Gamma_{m}C_{m}X)}, \end{array}$$

To update the precision matrix Γ for B fixed, we take advantage of the gradient spelt out in the online supplementary material. At a stationary point where $\nabla_{\Gamma }L(B,\Gamma )=0_{r\times r}$, the optimal Γ is


(4) $$\begin{array}{c} \nabla_{\Gamma }L(B,\Gamma )=\frac{n}{2}\Gamma^{-1}-\frac{1}{2}(Y-BX){\left(Y-BX\right)}^{T} \end{array}$$

(5) $$\begin{array}{c} \Gamma_{m+1}={\left[\frac{1}{n}(Y-B_{m}X){\left(Y-B_{m}X\right)}^{T}\right]}^{-1}. \end{array}$$

Equation (5) preserves the symmetry and positive semidefiniteness of $\Gamma_{m}$. The required matrix inversion is straightforward unless the number of traits *r* is exceptionally large. Our experiments suggest solving for $\Gamma_{m+1}$ exactly is superior to running full IHT jointly on both B and Γ. Algorithm 1 displays our block ascent algorithm.

### 2.2 Linear algebra with compressed genotype matrices

We previously described how to manipulate PLINK files using the OpenMendel module SnpArrays.jl (Zhou et al. 2020), which supports linear algebra on compressed genotype matrices (Chu et al. 2020). We now outline several enhancements to our compressed linear algebra routines.

**Compact genotype storage and fast reading.** A binary PLINK genotype (Purcell et al. 2007) stores each SNP genotype in two bits. Thus, an n×p genotype matrix requires 2*np* bits of memory. For bit-level storage Julia (Bezanson et al. 2017) supports the 8-bit unsigned integer type (UInt8) that can represent four sample genotypes simultaneously in a single 8-bit integer. Extracting sample genotypes can be achieved via bitshift and bitwise and operations. Genotypes are stored in little-endian fashion, with 0, 1, 2, and missing genotypes mapped to the bit patterns 00, 10, 11, and 01, respectively. For instance, if a locus has four sample genotypes 1, 0, 2, and missing, then the corresponding UInt8 integer is 01110010 in binary representation. Finally, because the genotype matrix is memory-mapped, opening a genotype file and accessing data are fast even for very large files.


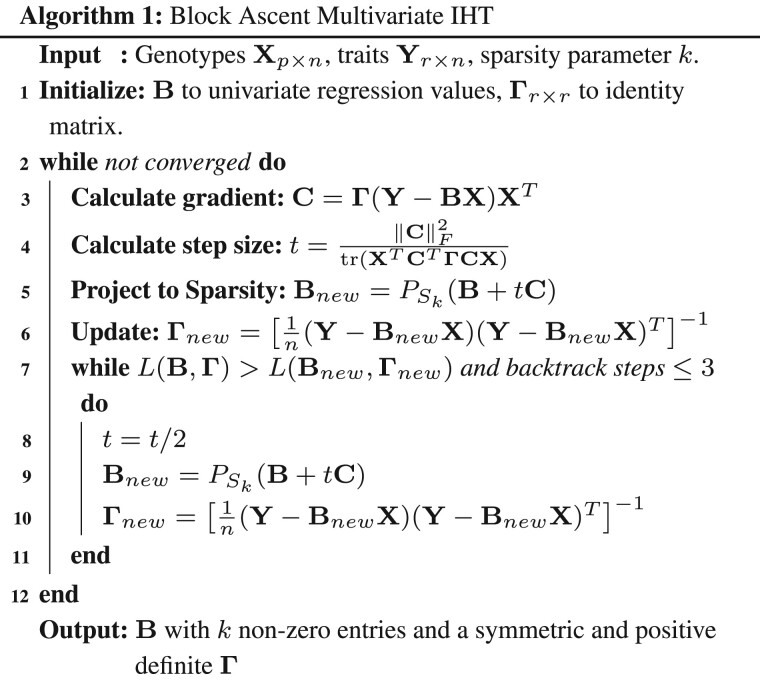


**Single instruction, multiple data (SIMD)-vectorized and tiled linear algebra.** In IHT, the most computationally intensive operations are the matrix-vector and matrix-matrix multiplications required in computing gradients. To accelerate these operations, we employ SIMD vectorization and tiling. On machines with SIMD support, such as Advanced Vector Extensions, our linear algebra routine on compressed genotypes is usually twice as fast as Basic Linear Algebra Subroutines (BLAS) 2 (Lawson et al. 1979) calls with an uncompressed numeric matrix and comparable in speed to BLAS 3 calls if B is tall or flat. These benchmarks are available on GitHub https://github.com/OpenMendel/SnpArrays.jl/blob/master/docs/SnpLinAlg.ipynb.

Computation of the matrix product C=AB requires special care when A is the binary PLINK-formatted genotype matrix and B and C are numeric matrices. The idea is to partition these three matrices into small blocks and exploit the representation $C_{ij}=\sum_{k}A_{ik}B_{kj}$ by computing each tiled product $A_{ik}B_{kj}$ in parallel. Because entries of a small matrix block are closer together in memory, this strategy improves cache efficiency. The triple for loops needed for computing products $A_{ik}B_{kj}$ are accelerated by invoking Julia’s LoopVectorization.jl package, which performs automatic vectorization on machines with SIMD support. Furthermore, this routine can be parallelized because individual blocks can be multiplied and added independently. Because multi-threading in Julia is composable, these parallel operations can be safely nested inside other multi-threading Julia functions, such as IHT’s cross-validation routine.

### 2.3 Simulated data experiments

Our simulation studies are based on the Chromosome 1 genotype data of the Northern Finland Birth Cohort (NFBC) (Sabatti et al. 2009). The original NFBC1966 data contain 5402 subjects and 364 590 SNPs; 26 906 of the SNPs reside on Chromosome 1. After filtering for subjects with at least 98% genotype success rate and SNPs with missing data <2%, we ended with 5340 subjects and 24 523 SNPs on Chromosome 1. For *r* traits, traits are simulated according to the matrix normal distribution (Dawid 1981, Yin and Li 2012, Furlotte and Eskin 2015) as using the OpenMendel module TraitSimulation.jl (Ji et al. 2021). Here, X is the Chromosome 1 NFBC p×n genotype matrix with *n* subjects aligned along its columns. The matrix B contains the true regression coefficients $b_{ij}$ uniformly drawn from {0.05,0.1,…,0.5} and randomly set to 0 so that $k_{\mathrm{true}}$ entries $b_{ij}$ survive. In standard mathematical notation, $||B|{|}_{0}=k_{\mathrm{true}}$. Note, the effect-size set {0.05,0.1,…,0.5} is comparable to previous studies (Chu et al. 2020). To capture pleiotropic effects, $k_{\mathrm{plei}}$ SNPs are randomly chosen to impact two traits. The remaining $k_{\mathrm{indep}}$ causal SNPs affect only one trait. Thus, $k_{\mathrm{true}}=2k_{\mathrm{plei}}+k_{\mathrm{indep}}$. Note, it is possible for two traits to share 0 pleiotropic SNPs. The row (trait) covariance matrix Σ is simulated so that its maximum condition number does not exceed 10. The column (sample) covariance matrix equals $\sigma_{g}^{2}\Phi +\sigma_{e}^{2}I$, where Φ is the centered genetic relationship matrix estimated by GEMMA (Zhou and Stephens 2014). We let $\sigma_{g}^{2}=0.1$ and $\sigma_{e}^{2}=0.9$. Different combinations of *r*, $k_{\mathrm{true}}$, $k_{\mathrm{indep}},$ and $k_{\mathrm{plei}}$ are summarized in Table 1. Each combination is replicated 100 times. It is worth emphasizing that this generative model should favor LMM analysis.


$$\begin{array}{l} Y_{r\times n}∼\text{MatrixNormal}(B_{r\times p}X_{p\times n},\;\Sigma_{r\times r},\;\sigma_{g}^{2}\Phi_{n\times n}+\sigma_{e}^{2}I_{n\times n}) \end{array}$$

**Table 1.** Comparison of mIHT and multiple uIHT implemented in MendelIHT, CAA implemented in mv-PLINK, and mvLMM implemented in GEMMA^a^.

	Time (s)	Plei power	Indep power	FP
Set 1: (2 traits, $k_{\mathrm{true}}=10,\;k_{\mathrm{indep}}=4,\;k_{\mathrm{plei}}=3$**)**				
mIHT	164.6±69.3	0.92±0.16	0.76±0.2	3.7±6.4
uIHT	114.9±48.6	0.93±0.16	0.72±0.2	1.4±3.7
CCA	152.6±57.3	0.96±0.14	0.78±0.2	77.8±0.2
mvLMM	307.7±121.4	0.95±0.15	0.76±0.2	42.8±18.5
Set 2: (3 traits, $k_{\mathrm{true}}=20,\;k_{\mathrm{indep}}=10,\;k_{\mathrm{plei}}=5$)				
mIHT	214.4±100.1	0.91±0.12	0.75±0.14	5.7±6.0
uIHT	169.6±81.9	0.86±0.16	0.72±0.16	2.4±2.5
CCA	226.8±101.9	0.95±0.09	0.79±0.15	125.3±55.3
mvLMM	449.9±221.7	0.93±0.1	0.75±0.16	66.1±22.8
Set 3: (5 traits, $k_{\mathrm{true}}=30,\;k_{\mathrm{indep}}=16,\;k_{\mathrm{plei}}=7$)				
mIHT	227.9±41.1	0.93±0.09	0.73±0.12	5.9±4.6
uIHT	213.8±45.7	0.90±0.11	0.69±0.12	3.2±3.8
CCA	371.5±34.0	0.96±0.07	0.75±0.11	173.2±54.9
mvLMM	1135.3±125.5	0.94±0.09	0.71±0.11	93.6±22.7
Set 4: (10 traits, $k_{\mathrm{true}}=10,\;k_{\mathrm{indep}}=4,\;k_{\mathrm{plei}}=3$)				
mIHT	278.8±53.0	0.97±0.09	0.74±0.20	2.2±2.0
uIHT	245.8±34.8	0.96±0.11	0.70±0.23	3.1±6.4
CCA	985.1±97.5	0.99±0.06	0.78±0.20	64.6±30.5
mvLMM	8067.4±3900.8	0.99±0.06	0.74±0.18	41.8±16.4
Set 5: (50 traits, $k_{\mathrm{true}}=20,\;k_{\mathrm{indep}}=10,\;k_{\mathrm{plei}}=5$)				
mIHT	1892.2±419.0	0.93±0.12	0.75±0.14	2.9±2.5
uIHT	1336.9±310.2	0.92±0.11	0.72±0.12	7.6±5.9
CCA	26 589.1±907.7(*)	NA	NA	NA
mvLMM	NA	NA	NA	NA
Set 6: (100 traits, $k_{\mathrm{true}}=30,\;k_{\mathrm{indep}}=16,\;k_{\mathrm{plei}}=7$)				
mIHT	3699.3±410.4	0.91±0.11	0.71±0.11	2.8±2.1
uIHT	2353.8±212.3	0.92±0.11	0.7±0.1	10.7±4.3
CCA	NA	NA	NA	NA
mvLMM	NA	NA	NA	NA

a Traits were simulated consistent with the Chromosome 1 SNPs of the NFBC1966 data. Plei power is power for pleiotopic SNPs, Indep power is power for independent SNPs, and FP is the total number of FPs, which potentially includes variants in high LD. Fig. 1 repeats the same simulation with LD-pruning. Displayed numbers are mean ± SDs. $k_{\mathrm{true}}$ is the total number of non-zero entries in B, $k_{\mathrm{indep}}$ is the number of independent SNPs affecting only one trait, and $k_{\mathrm{plei}}$ is the number of pleiotropic SNPs affecting two traits. These numbers satisfy $k_{\mathrm{true}}=2\times k_{\mathrm{plei}}+k_{\mathrm{indep}}$. Each simulation relied on 100 replicates. NA: >24 h. (*) Only two replicates contribute to timing.

Finally, using PLINK (Purcell et al. 2007), we generated three additional datasets by filtering out all SNPs whose pairwise correlation exceeds 0.25, 0.5, and 0.75. This action resulted in 7594, 13 441, and 18 580 SNPs, respectively. These reduced sets of data are used to study the effect of LD on power and FP rates in our subsequent comparisons of the competing methods.

### 2.4 Method comparisons

In our simulation experiments, we compared multivariate IHT (mIHT) to running multiple separate univariate IHT (uIHT) analyses (Keys et al. 2017, Chu et al. 2020), CCA implemented in mv-PLINK (Ferreira and Purcell 2009), and mvLMM implemented in GEMMA (Zhou and Stephens 2014). The LMM software GEMMA is broadly popular in genetic epidemiology. The software mv-PLINK is chosen for its speed. A recent review (Galesloot et al. 2014) rates it as the second fastest of the competing programs. The fastest method, mvBIMBAM (Stephens 2013), is an older method published by the authors of GEMMA, so it is not featured in this study.

In simulated data experiments, all programs were run within 16 cores of an Intel Xeon Gold 6140 2.30 GHz CPU with access to 32 GB of RAM. All experiments relied on version 1.4.2 of MendelIHT and Julia v1.5.4. IHT’s sparsity level *k* is tuned by cross-validation. The number of cross-validation paths is an important determinant of both computation time and accuracy. Thus, for simulated data, we employed an initial grid search involving 5-fold cross-validation over the sparsity levels k∈{5,10,…,50}. This was followed by 5-fold cross-validation for $k\in \{k_{\mathrm{best}}-4,\ldots ,k_{\mathrm{best}}+4\}$. This strategy first searches the space of potential values broadly, then, zooms in on the most promising candidate sparsity level. GEMMA and mv-PLINK were run under their default settings. For both programs, we declared SNPs significant whose *P*-values were lower than .05 divided by the number of SNPs tested. For GEMMA, we used the Wald test statistic.

### 2.5 Quality control for UK Biobank

We conducted two separate MendelIHT.jl analyses on the second release of the UK Biobank (Sudlow et al. 2015), containing ∼500 000 subjects and ∼800 000 SNPs. Our first analysis deals with three hypertension traits: average systolic blood pressure (SBP), average diastolic blood pressure (DBP), and body mass index (BMI). Our second analysis deals with 18 metabolomic quantitative traits related to total lipidomics.

All traits were first log-transformed to minimize the impact of skewness. Then each trait was standardized to mean 0 and variance 1, so that the traits were treated similarly in mean-squared error (MSE) cross-validation. Following Chu et al. (2020), German et al. (2020), and Ko et al. (2022a), we first filtered subjects exhibiting sex discordance, high heterozygosity, or high SNP missingness. We then excluded subjects of non-European ancestry and first and second-degree relatives based on empirical kinship coefficients. For three-trait hypertension analysis, we also excluded subjects on hypertension medicine at baseline. Finally, we excluded subjects with <98% genotyping success rate and SNPs with <99% genotyping success rate and imputed the remaining missing genotypes by the corresponding sample-mean genotypes. Note that imputation occurs in IHT on-the-fly.

The final dataset contains 470 228 SNPs and 185 656 subjects for the three hypertension traits and 104 264 subjects for the metabolomics traits. Given these reduced data and ignoring the Biobank’s precomputed principal components, we computed afresh the top 10 principal components of the genotype matrix via FlashPCA2 (Abraham et al. 2017) for the three-trait analysis and ProPCA (Agrawal et al. 2020) for the 18-trait analysis. These principal components serve as predictors to adjust for hidden ancestry. We also designated sex, age, and age^2^ as non-genetic predictors.

## 3 Results

### 3.1 Simulation experiments

Table 1 summarizes the various experiments conducted on the simulated data. For IHT, 5-fold cross-validation times are included. mIHT is the fastest method across the board and the only one that can analyze more than 50 traits. mIHT’s runtime increases roughly linearly with the number of traits and, as demonstrated previously, with sample size as well (Chu et al. 2020). All methods perform similarly in recovering the pleiotropic and independent SNPs. uIHT exhibits slightly worse true positive rate compared to multivariate methods. Given the identically distributed effect sizes in our simulations, all methods are better at finding pleiotropic SNPs than independent SNPs.

In Table 1, the number of FPs for both univariate and mIHT are much lower than competing methods. Presumably, many of the FPs from mvLMM and CCA represent SNPs in significant LD with the causal SNP. To study this phenomenon more closely, we repeated simulations in Sets 1–4 with LD-pruning of SNPs based on pairwise correlations (see Section 2 on how this is done prior to simulation). Figure 1 displays the number of FPs based on three separate LD-pruned datasets. Power comparison plots are available in the online supplementary material. IHT is better at distilling the true signal within these LD blocks, with or without LD-pruning, because IHT considers the effect of all SNPs jointly. Also mvLMM is better at controlling FPs than CCA, but mvLMM is slower, especially for large numbers of traits. In summary, IHT offers better model selection than its competitors with better computational speed.

**Figure 1:**
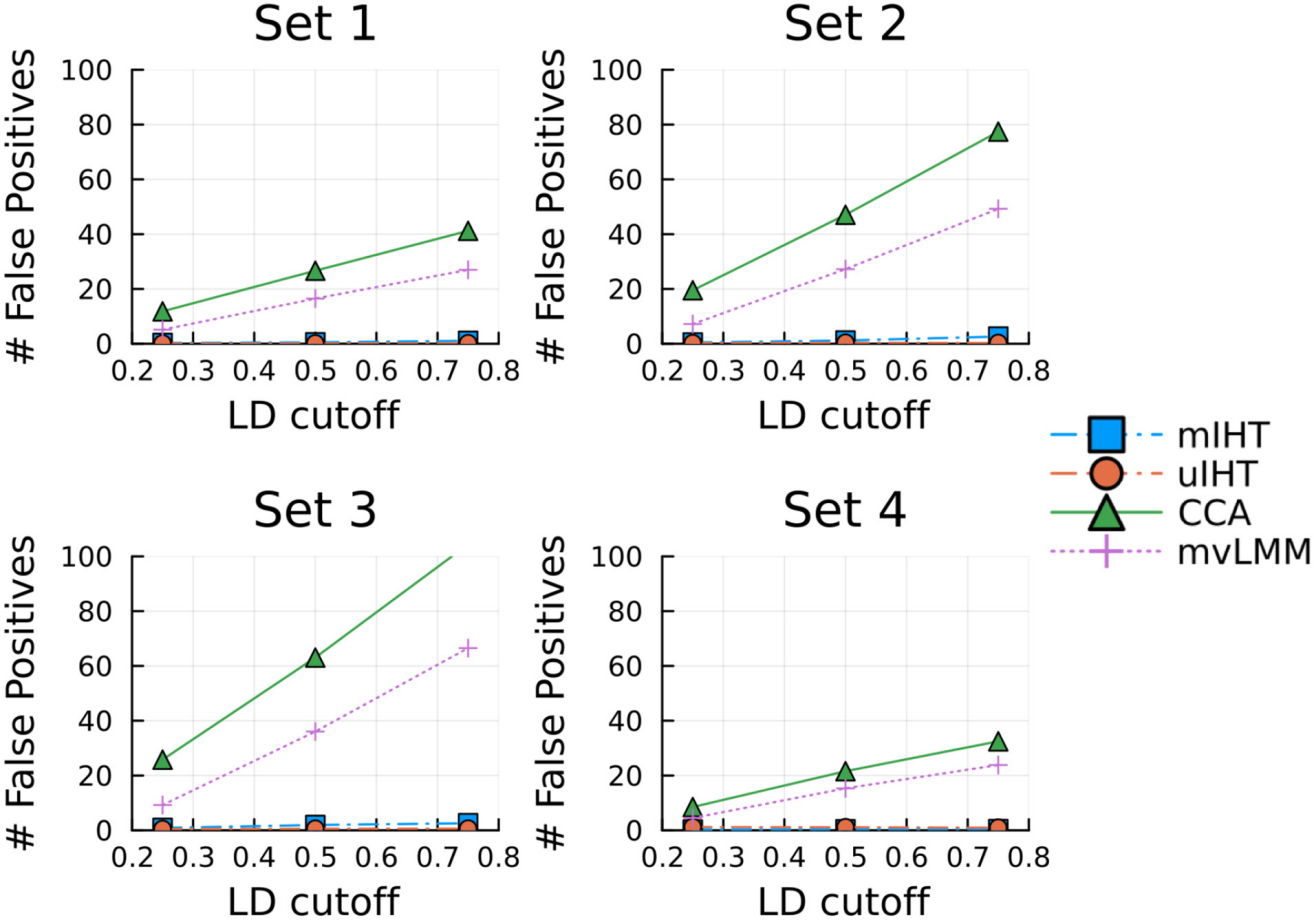
FP counts evaluated on LD-pruned genotypes reveal mIHT maintains low FP counts even on datasets that are in increasing linkage equilibrium. The *x*-axis corresponds to filtering the original NFBC chr1 genotypes at different pairwise correlation cutoffs. A smaller value means more aggressive pruning.

### 3.2 Three-trait UK Biobank analysis

With three hypertension traits, the UK Biobank analysis completed in 20 h and 8 min on 36 cores of an Intel Xeon Gold 6140 2.30 GHz CPU with access to 180 GB of RAM. As described in the methods section, the featured traits are BMI, average SBP, and average DBP. A first pass with 3-fold cross-validation across model sizes k∈{100,200,…,1000} showed that k=200 minimizes the MSE. A second pass with 3-fold cross-validation across model sizes k∈{110,120,…,290} showed that k=190 minimizes the MSE. A third 3-fold cross-validation pass across k∈{181,182,…,199} identified k=197 as the best sparsity level. Given k=197, we ran mIHT on the full data to estimate effect sizes, correlation among traits, and proportion of phenotypic variance explained by the genotypes.

IHT selected 13 pleiotropic SNPs and 171 independent SNPs. Selected SNPs and non-genetic predictors appear in Supplementary Tables S1–S5. To compare against previous studies, we used the R package gwasrapidd (Magno and Maia 2020) to search the NHGRI-EBI GWAS catalog (MacArthur et al. 2017) for previously associated SNPs within 1 Mb of each IHT discovered SNP. After matching, all 13 pleiotropic SNPs and 158 independent SNPs are either previously associated or are within 1 Mb of a previously associated SNP. We discovered 3 new associations with SBP and 10 new associations associated with DBP. Seven SNPs, rs2307111, rs6902725, rs11977526, rs2071518, rs11222084, rs365990, and rs77870048, are associated with two traits in opposite directions.

One can estimate the genotypic variance explained by the sparse model as $\text{Var}({\hat{\beta }}_{i}X)/\text{Var}(y_{i})$ for each trait $y_{i}$ where ${\hat{\beta }}_{i}\in R^{1\times p}$ is the *i*th row of B. MendelIHT.jl outputs the values $\sigma_{\mathrm{BMI}}^{2}=0.033$, $\sigma_{\mathrm{SBP}}^{2}=0.143$, and $\sigma_{\mathrm{DBP}}^{2}=0.048$. Note these estimates do not include contributions from the intercept or non-genetic predictors. The estimated correlations among traits are $r_{\mathrm{BMI},\mathrm{SBP}}=0.197$, $r_{\mathrm{BMI},\mathrm{DBP}}=0.286$, and $r_{\mathrm{SBP},\mathrm{DBP}}=0.738$. As expected, all traits are positively correlated, with a strong correlation between SBP and DBP and a weak correlation between BMI and both SBP and DBP.

### 3.3 18-Trait UK Biobank analysis

A separate analysis of the 18 UK Biobank lipid traits finished in 53 h on 32 cores of an AMD EPYC 7502P 2.5 GHz CPU with access to 252 GB of RAM. The peak RAM usage was 80.1 GB as measured by the seff command available on slurm clusters. Our cross-validation search started with an initial grid of k∈{1000,2000,…,10 000} and eventually terminated with k=4678. The IHT run-time script with its detailed cross-validation path is available in the online supplementary material.

**Figure 2:**
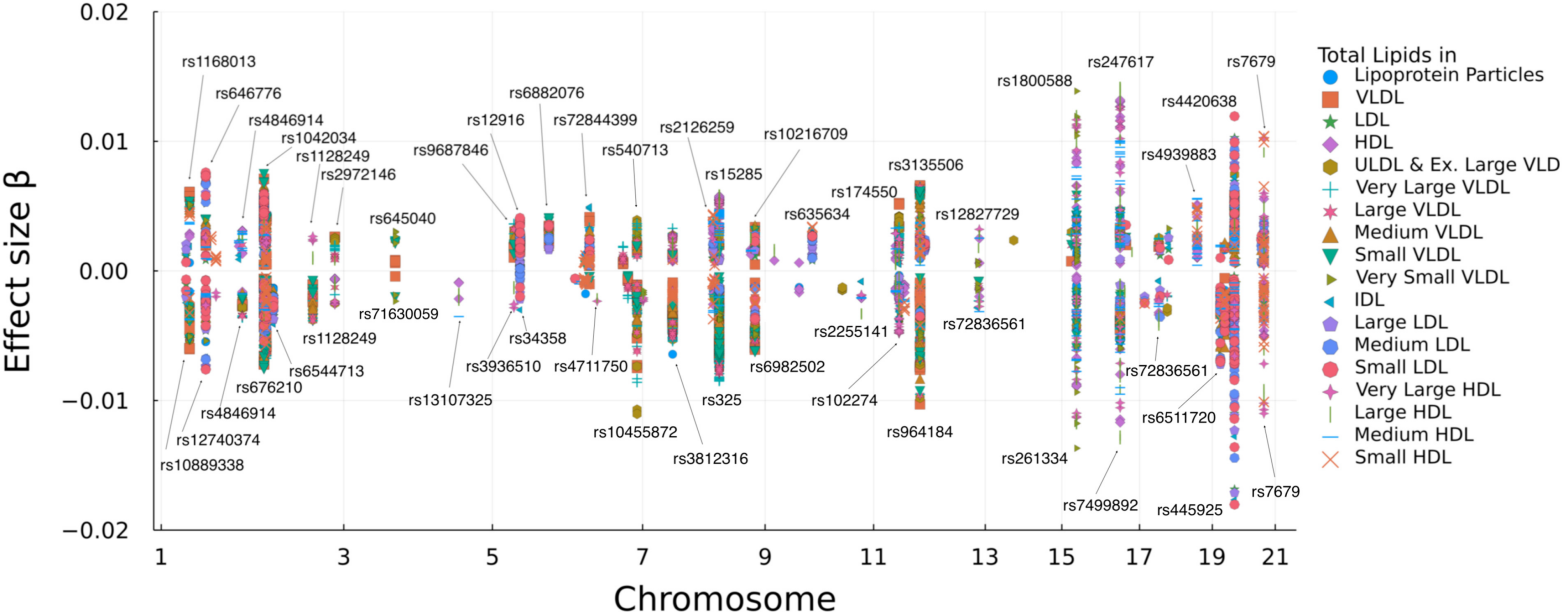
An 18-trait joint analysis on UK Biobank’s metabolomic traits using mIHT. The effect size for each trait is plotted against its chromosome position. The larger effect sizes are labeled with their SNP names. Note, one unit increase in effect size does not directly translate to one unit increase of lipids levels in its original scale because all traits were log-transformed and standardized. The featured metabolomic traits are available under category 220 of the UK Biobank where their field IDs appear in Supplementary Table S6.

mIHT found 218 independent and 699 pleiotropic SNPs for the 18 lipid traits. On average, a pleiotropic SNP is associated with 6.4 distinct lipid traits, suggesting that most significant SNPs for total lipid level are highly pleiotropic. Figure 2 depicts estimated effect sizes. The complete list of effect sizes as well as the estimated trait covariance matrix can be downloaded from our software page. The proportion of variance explained for each trait [roughly estimated as $\text{Var}({\hat{\beta }}_{i}X)/\text{Var}(y_{i})$] appears in the online supplementary material.

Although all traits are related to total lipids, we observe many associated genes containing distinct SNPs with opposite effects. Some of these reversals are caused by negatively correlated traits. Others are byproducts of IHT estimating the effect size of the alternate allele rather than that of the reference allele. Interestingly, SNP rs7679 has a large negative effect for Very Large HDL but a large positive effect for Small HDL, despite the fact that the two traits are positively correlated. To verify this phenomenon, we conducted 18 univariate regressions considering only rs7679 plus an intercept. The result confirmed that this SNP indeed affects the two traits in opposite directions. SNPs, such as rs7679, are interesting candidates for follow-up studies.

## 4 Discussion

This article presents mIHT for analyzing multiple correlated traits. In simulation studies, mIHT exhibits similar true positive rates, significantly lower FP rates, and better overall speed than LMMs and CCA. Computational time for mIHT increases roughly linearly with the number of traits. Since IHT is a multivariate regression method, the estimated effect size for each SNP is explicitly conditioned on other SNPs and non-genetic predictors. Analyzing three correlated UK Biobank traits with ∼200 000 subjects and ∼500 000 SNPs took 20 h on a single machine. A separate 18-trait analysis with ∼100 000 subjects and ∼500 000 SNPs took 53 h. IHT can output the correlation matrix and a rough estimate of the proportion of variance explained for the component traits. MendelIHT.jl also automatically handles various input formats (binary PLINK, BGEN, and VCF files) by calling the relevant OpenMendel packages. If binary PLINK files are used, MendelIHT.jl avoids decompressing genotypes to full numeric matrices.

MendelIHT.jl’s superior speed is partly algorithmic and partly due to software/hardware optimization. Internally, each iteration of mIHT requires a small r×r Cholesky factorization, where *r* is the number of traits. Each iteration also requires a dense matrix–matrix multiplication for computing gradients. For r≤100 featured in this study, the factorization is trivial to compute. To speed up matrix multiplication, we developed a parallelized, tiled, and SIMD-vectorized kernel that directly operates on binary PLINK files. This key innovation allows us to achieve performance near BLAS 3 calls without decompressing genotypes to numeric matrices. Because this kernel can be safely nested within IHT’s parallelized cross-validation step, we believe MendelIHT.jl is capable of utilizing hundreds of compute cores on a single machine.

IHT’s statistical and computational advantages come with limitations. For instance, it does not deliver *P*-values and ignores hidden and explicit relatedness. IHT can exploit principal components to adjust for ancestry, but PCA alone is insufficient to account for small-scale family structure (Price et al. 2010). To overcome this limitation, close relatives can be excluded from a study. Additional simulations summarized in Supplementary Table S7 also suggest that analyzing traits of vastly different polygenic heritability may lead to slightly inflated FP rates for the less polygenic traits. Thus, researchers may need to exercise caution when using mIHT for multiple traits when polygenic heritability differs by more than an order of magnitude. Although our simulation studies suggest the contrary, there is also the possibility that strong LD may confuse IHT. Finally, it is unclear how IHT will respond to wrongly imputed markers, extreme trait outliers, and the rare variants generated by sequencing. In spite of these qualms, the evidence presented here is persuasive about IHT’s potential for multivariate GWAS.

We will continue to explore improvements to IHT. Extension to non-Gaussian traits is hindered by the lack of flexible multivariate distributions with non-Gaussian margins. Cross-validation remains computationally intensive in tuning the sparsity level *k*. Although our vectorized linear algebra routine partially overcomes many of the computational barriers, we feel that further gains are possible through GPU computing (Zhou et al. 2010a, Ko et al. 2020, 2021, 2022b). In model selection, it may also be possible to control FDR better with statistical knockoff strategies (Barber and Candès 2015, Sesia et al. 2021), especially if traits of vastly varying polygenicity are being considered. Given IHT’s advantages, we recommend it for general use with the understanding that genetic epidemiologists respect its limitations and complement its application with standard univariate statistical analysis.

## Supplementary Material

btad193_Supplementary_Data

## Data Availability

The Northern Finland Birth Cohort 1966 (NFBC1966) was downloaded from dbGaP under dataset accession pht002005.v1.p1. UK Biobank data are retrieved under Project ID: 48152 and 15678.
