# Supplementary material for: Multivariate genome-wide association analysis by iterative hard thresholding
Source: Bioinformatics. 2023 Apr 17;39(4):btad193. doi: 10.1093/bioinformatics/btad193 (PMC10133532; doi:10.1093/bioinformatics/btad193)
Supplement: btad193_Supplementary_Data [file btad193_supplementary_data.pdf]

Supplement of Multivariate Genome-wide Association Analysis by Iterative Hard Thresholding

April 12, 2023

1 Supplementary Materials

1.1 SNPs Discovered by the UKB Analysis

Tables 1-5 list the SNPs discovered by our 3-trait UK Biobank analysis. For our 18-trait analysis the selected SNPs are too numerous to list. However, all result can be accessed from our software page. Table 6 lists the proportion of variance explained for the 18 traits. The reported effect sizes correspond to the predictors of the log-transformed and standardized traits. To compare against previous studies, we searched the NHGRI-EBI GWAS catalog [3] using the `R` package `gwasrapidd` [4]. For each SNP discovered by IHT, we queried a 1Mb radius for other SNPs that have been previously associated with the given trait with p value $< 5 \times 10^{-8}$. Each known association is defined by the most significant SNP in the gene or region associated with the trait. SNPs that have been mapped in the GWAS catalog are listed along with their genes. All 13 pleiotropic SNPs were previously known, and 158 out of 171 independent SNPs were previously known. Among the 13 newly discovered associations, three were with SBP and 10 were with DBP.

SNP	chr	pos	BMI	SBP	DBP	# prior report	mapped genes
rs1801131	1	11854476	0.0	0.009	0.008	41	MTHFR
rs17367504	1	11862778	0.0	0.02	0.019	41	MTHFR
rs16998073	4	81184341	0.0	-0.023	-0.023	24	FGF5, PRDM8
rs1173727	5	32830521	0.0	0.016	0.017	19	LINC02120, NPR3
rs2307111	5	75003678	0.013	0.0	-0.01	34	POC5
rs6902725	6	152370868	0.0	-0.009	0.01	4	
rs11977526	7	46008110	0.0	0.013	-0.011	6	FTLP15, IGFBP3
rs2071518	8	120435812	0.0	-0.008	0.009	6	CCN3
rs11222084	11	130273230	0.0	-0.011	0.009	9	ZBTB44-DT
rs3184504	12	111884608	0.0	-0.011	-0.018	41	SH2B3, ATXN2
rs365990	14	23861811	0.0	0.008	-0.011	6	MYH6
rs7497304	15	91429176	0.0	-0.019	-0.018	14	FES
rs77870048	16	69965021	0.0	-0.011	0.01	20	WWP2

Table 1: 13 pleiotropic SNPs selected by IHT listed with their effect sizes and sorted by their position on the chromosomes. An effect size of 0 means the particular predictor was not selected. The field *prior reports* records the number of SNPs previously associated with BMI, SBP, or DBP (p value < 10^{-8}) that are within 1Mb of the given SNP. BMI = body mass index; SBP = systolic blood pressure; DBP = diastolic blood pressure.

covariate	BMI	SBP	DBP
intercept	0.0002	-0.0	0.0003
sex	0.1165	0.1639	0.1808
age	0.1073	-0.1395	0.6484
age^2	-0.0505	0.4581	-0.6131
PC1	-0.0287	-0.0066	-0.0074
PC2	0.0052	-0.0077	-0.0011
PC3	0.0174	0.0043	-0.0091
PC4	-0.0019	-0.0055	0.0015
PC5	0.0022	0.0137	0.0167
PC6	-0.0165	-0.0067	-0.0056
PC7	-0.003	-0.0038	-0.0078
PC8	-0.0014	-0.0015	-0.0014
PC9	0.0039	-0.0004	0.0017
PC10	0.0068	0.0005	0.0001

Table 2: Non-genetic covariates estimated by IHT listed with their effect sizes. These variables were not subject to sparsity projection. PC is short for principal component. BMI = body mass index, SBP = systolic blood pressure, and DBP = diastolic blood pressure.

SNP	chr	pos	β	# prior reports	mapped genes	SNP	chr	pos	β	# prior reports	mapped genes
rs2815757	1	72764289	0.019	14	RPL31P12, NEGR1	rs17207196	7	75101065	0.016	9	POM121C
rs543874	1	177889480	-0.028	22	SEC16B, LINC01741	rs925946	11	27667202	-0.007	24	BDNF-AS
rs2820312	1	201869257	-0.014	9	LMOD1	rs6265	11	27679916	0.012	24	BDNF-AS, BDNF
rs62106258	2	417167	0.027	51		rs2049045	11	27694241	0.007	24	BDNF-AS, BDNF
rs11127485	2	632028	0.017	51	TMEM18, LINC01875	rs10835211	11	27701365	-0.011	24	BDNF-AS, BDNF
rs13393304	2	637830	0.008	51	TMEM18, LINC01875	rs7138803	12	50247468	-0.007	9	RPL35AP28, BCDIN3D
rs713586	2	25158008	-0.026	26	DNAJC27, ADCY3	rs7132908	12	50263148	-0.014	9	FAIM2
rs9821675	3	49902544	0.007	20		rs4776970	15	68080886	0.01	17	MAP2K5
rs1062633	3	49924940	0.012	20		rs16951304	15	68089618	0.008	17	
rs957919	3	131629716	-0.015	17		rs2531995	16	4013467	0.016	17	ADCY9
rs61587156	3	185831583	0.017	9		rs72793809	16	28832382	-0.015	17	
rs34811474	4	25408838	0.015	3	ANAPC4	rs4788190	16	29948401	0.019	16	
rs10938397	4	45182527	-0.022	17	PRDX4P1, THAP12P9	rs4889490	16	30823047	0.014	20	ZNF629, Metazoa_SRP
rs13107325	4	103188709	-0.026	7		rs1421085	16	53800954	-0.05	56	FTO
rs2112347	5	75015242	0.009	22	SLC25A5P9, POC5	rs17782313	18	57851097	-0.011	47	RNU4-17P, MC4R
rs1422192	5	87959023	-0.015	20		rs10871777	18	57851763	-0.015	47	RNU4-17P, MC4R
rs2744962	6	34594440	-0.017	33		rs17773430	18	57963117	-0.014	45	MC4R
rs987237	6	50803050	-0.012	37	TFAP2B	rs1800437	19	46181392	0.017	20	GIPR
rs9473932	6	50857995	-0.009	37	FTH1P5, RPS17P5	rs3810291	19	47569003	0.022	12	ZC3H4

Table 3: 38 SNPs associated with BMI independently of SBP and DBP listed with their effect sizes and sorted by their position on the chromosomes. The field *prior reports* records the number of GWAS Catalog associations with BMI (p value $< 10^{-8}$) that are within 1Mb of the given SNP.

SNP	chr	pos	β	# prior reports	mapped genes	SNP	chr	pos	β	# prior reports	mapped genes
rs3936009	1	1585642	0.009	7		rs12673516	7	40432219	0.01	3	
rs1757915	1	56615809	-0.009	4	RPSAP20, LINC01755	rs2282978	7	92264410	0.016	4	CDK6
rs6684353	1	59638619	0.011	2		rs2392929	7	106414069	-0.027	5	LINC02577, CCDC71L
rs12069946	1	67045928	-0.009	2		rs2978456	8	42324765	-0.01	1	
rs2820441	1	219734960	0.009	1	LYPLAL1-AS1, ZC3H11B	Affx-32837790	8	95272605	-0.009	3	
rs1522484	2	19708967	0.016	3		rs35758124	8	141048964	-0.01	2	
rs9306894	2	20878105	0.009	4	GDF7	rs10757278	9	22124477	-0.01	3	CDKN2B-AS1
rs55654088	2	85499562	-0.01	4		rs10986626	9	127948321	-0.009	5	
rs13002573	2	164915208	0.012	13	intergenic	rs12258967	10	18727959	0.009	10	CACNB2
rs560887	2	169763148	0.011	1	SPC25, G6PC2	rs1908339	10	75843100	0.01	4	
rs10497529	2	179839888	0.011	3	CCDC141	rs11191064	10	103367824	0.009	6	
rs1052501	3	41925398	0.02	6	ULK4	rs11598702	10	104897985	-0.011	17	
rs2498323	4	3451109	-0.008	3	HGFAC	rs4980389	11	1892585	-0.009	14	LSP1
rs776590	4	48591150	0.008	2		rs573455	11	117267884	-0.014	6	CEP164
rs17084051	4	55087581	-0.01	3	RPL22P13	rs10750441	11	130469044	0.01	3	BAK1P2, MIR8052
rs1229984	4	100239319	0.009	1	ADH1B	rs10770612	12	20230639	0.012	9	LINC02398
rs6842241	4	148400819	-0.008	2	EDNRA, PRMT5P1	rs2681492	12	90013089	0.011	21	ATP2B1
rs4690974	4	156393641	-0.01	9	MTND1P22	rs12882307	14	36320639	0.009	1	
rs13116200	4	169702511	-0.01	2		rs4903064	14	73279420	-0.01	5	DPF3
rs7715779	5	32690199	0.007	12	NPR3	rs956006	15	62808539	0.009	1	TLN2
rs1173771	5	32815028	0.005	12	LINC02120, NPR3	rs34862454	15	75101530	-0.012	14	LMAN1L, CSK
rs1982192	5	71540604	0.009	Novel		rs3803716	16	24802325	0.008	3	
rs2303720	5	122682334	0.011	6	CEP120	rs4888372	16	75313485	0.01	6	RNU6-758P, BCAR1
rs11954193	5	158256118	0.01	13	EBF1	rs60675007	16	83018052	0.008	2	
rs12198986	6	7720059	-0.008	1	BMP6	rs9889363	17	6524298	-0.011	6	
rs9349379	6	12903957	0.012	5	PHACTR1	rs185478092	17	40366653	-0.01	Novel	
rs385306	6	31681160	0.011	7		rs3744760	17	43195981	-0.01	9	PLCD3
rs12191865	6	56014006	-0.014	3		rs17608766	17	45013271	-0.013	3	GOSR2
rs1012257	6	56089507	-0.009	3		rs9909933	17	46280232	0.009	7	
rs9689048	6	73465420	0.009	Novel		rs35688424	17	60769406	-0.011	8	
rs2221389	6	159719183	-0.012	1		rs67882421	18	43129717	-0.012	4	
rs9505897	6	169646751	-0.01	1		rs12459507	19	2224387	-0.009	6	DOT1L
rs57301765	7	19052733	-0.014	2	TWIST1, HDAC9	rs34328549	19	7253184	0.007	8	INSR

Table 4: 66 SNPs associated with SBP independently of BMI and DBP listed with their effect sizes and sorted by their position on the chromosomes. The field *prior reports* records the number of GWAS Catalog associations with SBP (p value < 10^{-8}) that are within 1Mb of the given SNP. A novel SNP is not within 1Mb of any GWAS Catalog associations.

SNP	chr	pos	β	# Prior reports	mapped genes	SNP	chr	pos	β	# Prior reports	mapped genes
rs61776719	1	38461319	-0.015	Novel	FHL3, RNU6-510P	rs4754834	11	102017853	-0.009	1	
rs12739904	1	59669488	-0.009	1		rs59317921	11	130270855	0.009	2	ZBTB44-DT
rs3766090	1	169269009	-0.01	Novel		rs7975252	12	15270194	-0.009	1	
rs61822997	1	176649785	-0.009	Novel		rs7973748	12	20164705	0.011	10	
rs665834	1	201748087	0.011	1		rs12581906	12	20468094	0.009	10	
rs2275155	1	243493907	-0.013	6		rs17287293	12	24770878	0.01	Novel	RN7SL38P, KNOP1P1
rs11690961	2	46363336	-0.012	1	PRKCE	rs74340001	12	94769769	-0.01	1	
rs10199082	2	56040099	-0.01	3		rs653178	12	112007756	-0.007	16	ATXN2
rs2692893	2	96756547	-0.012	5		rs12875271	13	110792743	0.011	3	RN7SL783P, COL4A1
rs17362588	2	179721046	-0.014	3	CCDC141	rs36033161	14	100123487	-0.009	Novel	HHIPL1
rs12996836	2	183212028	-0.011	3		rs686861	15	48885005	-0.013	1	FBN1
rs1863703	2	219544388	0.009	3		rs11853359	15	71621524	0.011	2	THSD4
rs2624847	3	50174197	-0.009	3	SEMA3F-AS1	rs1378942	15	75077367	-0.012	10	CSK
rs3617	3	52833805	0.008	5	ITIH3	rs4886615	15	75131661	-0.008	10	
rs9850919	3	169177924	0.009	5	MECOM-AS1, MECOM	rs2277547	15	79082431	0.01	1	
rs871606	4	54799245	-0.018	1	RPL21P44, LNX1	rs7174546	15	96637569	-0.009	2	RNU2-3P, NR2F2-AS1
rs1826909	4	100217743	-0.01	Novel		rs67456613	16	30888295	-0.01	2	
rs1047440	5	122681834	-0.014	3		rs72790195	16	83005335	-0.008	1	
rs11949055	5	131576737	0.009	5		rs11078485	17	4141536	-0.009	2	
rs1233708	6	28173219	0.013	2		rs72824497	17	15418019	0.009	1	
rs11154022	6	121748542	0.01	3	GJA1, Y_RNA	rs768168	19	15268533	-0.011	Novel	
rs12110693	6	122158270	-0.012	3	OSTM1, HMGB3P18	rs997669	19	30304483	-0.01	2	CCNE1
rs9376740	6	143617983	-0.009	Novel		rs755690	19	39167360	-0.01	1	
rs58023137	6	169620760	-0.011	1		rs2876201	20	10429851	-0.009	6	
rs194524	7	89861832	-0.012	Novel		rs652661	20	10468779	0.015	6	
rs13226502	7	100506381	-0.014	2	RPS29P15, RN7SL549P	rs78309244	20	10766399	0.014	6	
rs2469997	8	120353267	0.01	3	CCN3, MIR548AZ	rs6046144	20	19477390	0.015	2	
rs507666	9	136149399	0.012	2		rs76701589	20	39763115	0.009	3	
rs3812595	9	139369062	-0.009	1	SEC16A	rs2831969	21	30085640	0.009	Novel	
rs183348357	10	18220414	-0.009	7		rs9982601	21	35599128	-0.009	1	LINC00310, KCNE2
rs3739998	10	30316072	0.005	1	JCAD	rs2298336	21	39813287	0.01	1	
rs2505083	10	30335122	0.007	1	JCAD	rs112005532	21	39957981	0.012	1	ERG
rs7125196	11	61272565	0.01	5	PPP1R32, MIR4488	rs73167017	22	40779964	0.011	1	SGSM3, SGSM3-AS1, ADSL
rs2304500	11	89223931	-0.012	1	NOX4						

Table 5: 67 SNPs associated with DBP independently of BMI and SBP listed with their effect sizes and sorted by their positions on the chromosomes. The field *prior reports* records the number of GWAS Catalog associations with DBP (p value $< 10^{-8}$) that are within 1Mb of the given SNP. A novel SNP is not within 1Mb of any GWAS Catalog associations.

Field ID	Trait	PVE
23723	Total Lipids in Lipoprotein Particles	0.0178
23724	Total Lipids in VLDL	0.0423
23725	Total Lipids in LDL	0.0245
23726	Total Lipids in HDL	0.0365
23782	Total Lipids in Chylomicrons and Extremely Large VLDL	0.0482
23789	Total Lipids in Very Large VLDL	0.0547
23796	Total Lipids in Large VLDL	0.0501
23803	Total Lipids in Medium VLDL	0.0332
23810	Total Lipids in Small VLDL	0.0432
23817	Total Lipids in Very Small VLDL	0.0370
23824	Total Lipids in IDL	0.0268
23831	Total Lipids in Large LDL	0.0250
23838	Total Lipids in Medium LDL	0.0230
23845	Total Lipids in Small LDL	0.0268
23852	Total Lipids in Very Large HDL	0.0515
23859	Total Lipids in Large HDL	0.0538
23866	Total Lipids in Medium HDL	0.0268
23873	Total Lipids in Small HDL	0.0136

Table 6: The estimated Proportion of phenotypic variance explained (PVE) for each of the 18 traits in the UK Biobank analysis.

1.2 IHT on LD-Pruned Genotypes

In Figure 1 of the main text, we compared the false positive counts of IHT, CCA, and mvLMM. Similar to Table 1 in the main text, we also computed the power for detecting independent and pleiotropic SNPs in these simulations. These results are presented in Figure 1.

1.3 Additional Simulation Studies for IHT

In the main text, our simulation study explores the situation where causal SNPs are shared roughly equally among all traits. This is a reasonable assumption because we expect most multivariate GWAS to be conducted on similar traits, which are expected to share a similar number of causal variants. However, in practice, researchers may confront both highly polygenic traits along with very non-polygenic traits.

Here we describe a simulation study with three traits, where each trait is perturbed by 10, 100, or 1000 causal SNPs, respectively. We use $n = 10,000$ unrelated subjects from the UK Biobank and restrict analysis to a genotype matrix constructed from $29,481$ SNPs on chromosome 10. Traits $\mathbf{Y}_{r\times 10000}$ are simulated just as in our main simulation study. We ignore the genetic relationship matrix since related subjects have been

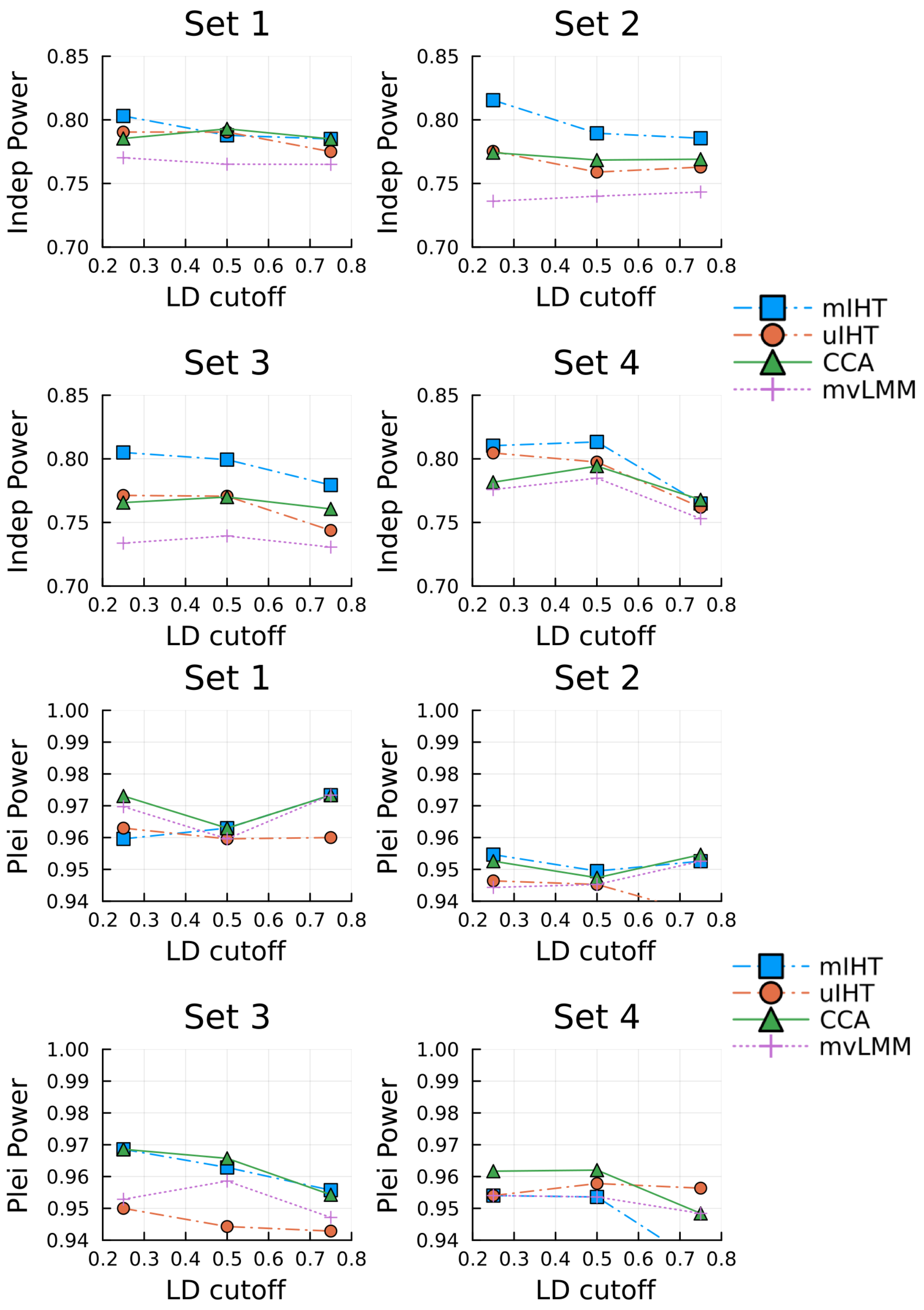

Figure 1: Power comparison for independent (top 4) and pleiotropic (bottom 4) SNPs evaluated on LD-pruned genotypes that are in increasing linkage equilibrium. The x-axis corresponds to filtering the original NFBC chr1 genotypes at different pairwise correlation cutoffs, a smaller value means more aggressive pruning.

	Power	FDR
Trait 1 ($k = 10$)	0.417	0.408
Trait 2 ($k = 100$)	0.316	0.385
Trait 3 ($k = 1000$)	0.250	0.362
Overall	0.258	0.366

Table 7: Multivariate IHT analyzing three simulated traits of different polygenicity. The three traits have 10, 100, and 1000 causal SNPs.

filtered out. In summary,

$$\mathbf{Y}_{3\times n} \quad \sim \quad \text{MatrixNormal}(\mathbf{B}_{3\times p}\mathbf{X}_{p\times n},\ \ \boldsymbol{\Sigma}_{3\times 3},\ \ \mathbf{I}_{n\times n}).$$

For the rth trait, $r \in \{1,2,3\}$, the effect sizes of the causal SNPs $j \in \mathcal{S}_{r,\text{causal}}$ are Gaussian deviates $\beta_j \sim N(0,0.1)$ with $|\mathcal{S}_{1,\text{causal}}| = 10$, $|\mathcal{S}_{2,\text{causal}}| = 100$, and $|\mathcal{S}_{3,\text{causal}}| = 1000$. The causal SNP indices are chosen uniformly across the chromosome. The covariance between traits, $\boldsymbol{\Sigma}_{3\times 3}$, is generated as in our main simulation.

Table 7 reports the power and false discovery rate (FDR) for each trait separately, along with the overall power and FDR. Observe that FDR decreases as the number of causal SNPs k increases. Notably mIHT tends to select more SNPs than needed for a less polygenic trait if it is analyzed in unison with a highly polygenic trait. Obviously, this bias improves if we increase sample size or true effect sizes. Overall, these results suggest that one must be careful in analyzing multiple traits of vastly different polygenicity.

1.4 Loglikelihood

Consider multivariate linear regression with r traits under a Gaussian model. Up to a constant, the loglikelihood for the response vector $\mathbf{y}_i$ of subject i can be written

$$\begin{aligned}
\mathcal{L}_i(\mathbf{B},\boldsymbol{\Gamma}) &= \frac{1}{2}\log(\det\boldsymbol{\Gamma}) - \frac{1}{2}(\mathbf{y}_i - \mathbf{B}\mathbf{x}_i)^T\boldsymbol{\Gamma}(\mathbf{y}_i - \mathbf{B}\mathbf{x}_i) \\
&= \frac{1}{2}\log(\det\boldsymbol{\Gamma}) - \frac{1}{2}\operatorname{tr}[\boldsymbol{\Gamma}(\mathbf{y}_i - \mathbf{B}\mathbf{x}_i)(\mathbf{y}_i - \mathbf{B}\mathbf{x}_i)^T],
\end{aligned}$$

where $\mathbf{B}$ is the $r \times p$ matrix of regression coefficients, $\mathbf{x}_i$ is the $p \times 1$ vector of predictors, and $\boldsymbol{\Gamma}$ is the $r \times r$ unstructured precision (inverse covariance) matrix. For n independent subjects, let $\mathbf{Y}$ be the $r \times n$ matrix with ith column $\mathbf{y}_i$ and let $\mathbf{X}$ be the $p \times n$ design matrix with ith column $\mathbf{x}_i$. Then the loglikelihood for all subjects is

$$\begin{aligned}
\mathcal{L}(\mathbf{B},\boldsymbol{\Gamma}) &= \sum_{i=1}^{n}\left\{\frac{1}{2}\log(\det\boldsymbol{\Gamma}) - \frac{1}{2}\operatorname{tr}[\boldsymbol{\Gamma}(\mathbf{y}_i - \mathbf{B}\mathbf{x}_i)(\mathbf{y}_i - \mathbf{B}\mathbf{x}_i)^T]\right\} \\
&= \frac{n}{2}\log(\det\boldsymbol{\Gamma}) - \frac{1}{2}\operatorname{tr}\left[\boldsymbol{\Gamma}\sum_{i=1}^{n}(\mathbf{y}_i - \mathbf{B}\mathbf{x}_i)(\mathbf{y}_i - \mathbf{B}\mathbf{x}_i)^T\right] \\
&= \frac{n}{2}\log(\det\boldsymbol{\Gamma}) - \frac{1}{2}\operatorname{tr}[(\boldsymbol{\Gamma}(\mathbf{Y} - \mathbf{B}\mathbf{X})(\mathbf{Y} - \mathbf{B}\mathbf{X})^T].
\end{aligned}$$

In subsequent sections we will present both full and block ascent IHT. The former updates $\mathbf{B}$ and $\mathbf{\Gamma}$ simultaneously. The latter alternates updates of $\mathbf{B}$ and $\mathbf{\Gamma}$, holding the other parameter block fixed.

1.5 First Directional Derivative

Recall that the Hadamard's semi-directional derivative [1, 2] of a function $f(\mathbf{x})$ in the direction $\mathbf{v}$ is defined as the limit

$$d_{\mathbf{v}} f(\mathbf{x}) = \lim_{\substack{h \to 0 \\ \mathbf{w} \to \mathbf{v}}} \frac{f(\mathbf{x}+h\mathbf{w}) - f(\mathbf{x})}{h}.$$

To calculate the directional derivative of the loglikelihood (eq 1 in main text), we perturb $\mathbf{B}$ in the direction $\mathbf{U}$ and $\mathbf{\Gamma}$ in the symmetric direction $\mathbf{V}$. The sum and product rules then give

$$\begin{aligned} & d_{(\mathbf{U},\mathbf{V})} \operatorname{tr}[\mathbf{\Gamma}(\mathbf{Y}-\mathbf{B}\mathbf{X})(\mathbf{Y}-\mathbf{B}\mathbf{X})^T] \\ = \; & \operatorname{tr}[\mathbf{V}(\mathbf{Y}-\mathbf{B}\mathbf{X})(\mathbf{Y}-\mathbf{B}\mathbf{X})^T] - \operatorname{tr}[\mathbf{\Gamma}(\mathbf{Y}-\mathbf{B}\mathbf{X})\mathbf{X}^T\mathbf{U}^T + \mathbf{\Gamma}\mathbf{U}\mathbf{X}(\mathbf{Y}-\mathbf{B}\mathbf{X})^T]. \end{aligned}$$

The directional derivative $d_{\mathbf{V}} \log\det(\mathbf{\Gamma}) = \operatorname{tr}(\mathbf{\Gamma}^{-1}\mathbf{V})$ is derived in Example 3.2.6 of [2]. The trace properties $\operatorname{tr}(\mathbf{C}\mathbf{D}) = \operatorname{tr}(\mathbf{D}\mathbf{C})$ and $\operatorname{tr}(\mathbf{C}^T) = \operatorname{tr}(\mathbf{C})$ consequently imply

$$d_{(\mathbf{U},\mathbf{V})}\mathcal{L}(\mathbf{B},\mathbf{\Gamma}) = \frac{n}{2}\operatorname{tr}(\mathbf{\Gamma}^{-1}\mathbf{V}) - \frac{1}{2}\operatorname{tr}[(\mathbf{Y}-\mathbf{B}\mathbf{X})(\mathbf{Y}-\mathbf{B}\mathbf{X})^T\mathbf{V}] + \operatorname{tr}[\mathbf{X}(\mathbf{Y}-\mathbf{B}\mathbf{X})^T\mathbf{\Gamma}\mathbf{U}]. \tag{1.1}$$

Because this last expression is linear in $(\mathbf{U},\mathbf{V})$, the loglikelihood is continuously differentiable.

1.6 Second Directional Derivative

Now we take the directional derivative of the directional derivative (1.1) in the new directions $\tilde{\mathbf{U}}$ and $\tilde{\mathbf{V}}$. This action requires the inverse rule $d_{\tilde{\mathbf{V}}}\mathbf{\Gamma}^{-1} = -\mathbf{\Gamma}^{-1}\tilde{\mathbf{V}}\mathbf{\Gamma}^{-1}$ proved in Example 3.2.7 of [2]. Accordingly, we find

$$d_{(\tilde{\mathbf{U}},\tilde{\mathbf{V}})}\frac{n}{2}\operatorname{tr}(\mathbf{\Gamma}^{-1}\mathbf{V}) = -\frac{n}{2}\operatorname{tr}\left(\mathbf{\Gamma}^{-1}\tilde{\mathbf{V}}\mathbf{\Gamma}^{-1}\mathbf{V}\right).$$

We also calculate

$$d_{(\tilde{\mathbf{U}},\tilde{\mathbf{V}})}\left[-\frac{1}{2}\operatorname{tr}[(\mathbf{Y}-\mathbf{B}\mathbf{X})(\mathbf{Y}-\mathbf{B}\mathbf{X})^T\mathbf{V}]\right] = \frac{1}{2}\operatorname{tr}[(\mathbf{Y}-\mathbf{B}\mathbf{X})\mathbf{X}^T\tilde{\mathbf{U}}^T\mathbf{V}] + \frac{1}{2}\operatorname{tr}[\tilde{\mathbf{U}}\mathbf{X}(\mathbf{Y}-\mathbf{B}\mathbf{X})^T\mathbf{V}]$$

and

$$d_{(\tilde{\mathbf{U}},\tilde{\mathbf{V}})}\operatorname{tr}[\mathbf{X}(\mathbf{Y}-\mathbf{B}\mathbf{X})^T\mathbf{\Gamma}\mathbf{U}] = \operatorname{tr}[\mathbf{X}(\mathbf{Y}-\mathbf{B}\mathbf{X})^T\tilde{\mathbf{V}}\mathbf{U}] - \operatorname{tr}(\mathbf{X}^T\tilde{\mathbf{U}}^T\mathbf{\Gamma}\mathbf{U}\mathbf{X}).$$

Finally, setting the two directions equal so that $\tilde{\mathbf{V}} = \mathbf{V}$ and $\tilde{\mathbf{U}} = \mathbf{U}$ produces the quadratic form

$$\begin{aligned} Q(\mathbf{U},\mathbf{V}) = \; & -\frac{n}{2}\operatorname{tr}[\mathbf{\Gamma}^{-1}\mathbf{V}\mathbf{\Gamma}^{-1}\mathbf{V}] + \frac{1}{2}\operatorname{tr}[(\mathbf{Y}-\mathbf{B}\mathbf{X})\mathbf{X}^T\mathbf{U}^T\mathbf{V}] + \frac{1}{2}\operatorname{tr}[\mathbf{U}\mathbf{X}(\mathbf{Y}-\mathbf{B}\mathbf{X})^T\mathbf{V}] \\ & + \operatorname{tr}[\mathbf{X}(\mathbf{Y}-\mathbf{B}\mathbf{X})^T\mathbf{V}\mathbf{U}] - \operatorname{tr}(\mathbf{X}^T\mathbf{U}^T\mathbf{\Gamma}\mathbf{U}\mathbf{X}). \end{aligned} \tag{1.2}$$

generated by the second differential.

1.7 Extraction of the Gradient and Expected Information

To extract the gradient from a directional derivative, we recall the identity $d_{\mathbf{v}} f(\mathbf{x}) = \nabla f(\mathbf{x})^T \mathbf{v}$ for vectors $\mathbf{v}$ and $\mathbf{x}$ and the identity $\operatorname{tr}(\mathbf{A}^T\mathbf{B}) = \operatorname{vec}(\mathbf{A})^T \operatorname{vec}(\mathbf{B})$ for matrices $\mathbf{A}$ and $\mathbf{B}$ [5]. The first identity shows that the directional derivative is the inner product of the gradient with respect to the direction $\mathbf{v}$. The second displays the trace function as an inner product on dimensionally identical matrices. Thus, the matrix directional derivative is

$$d_{\mathbf{V}} f(\mathbf{X}) \quad = \quad \operatorname{vec}[\nabla f(\mathbf{X})]^T \operatorname{vec}(\mathbf{V}) \quad = \quad \operatorname{tr}[\nabla f(\mathbf{X})^T \mathbf{V}]. \tag{1.3}$$

Inspection of the directional derivative (1.1) now leads to the gradient with blocks

$$\begin{aligned} \nabla_{\mathbf{B}} \mathcal{L}(\mathbf{B},\mathbf{\Gamma}) &= [\mathbf{X}(\mathbf{Y}-\mathbf{B}\mathbf{X})^T\mathbf{\Gamma}]^T \quad = \quad \mathbf{\Gamma}(\mathbf{Y}-\mathbf{B}\mathbf{X})\mathbf{X}^T & (1.4) \\ \nabla_{\mathbf{\Gamma}} \mathcal{L}(\mathbf{B},\mathbf{\Gamma}) &= \frac{n}{2}\mathbf{\Gamma}^{-1} - \frac{1}{2}(\mathbf{Y}-\mathbf{B}\mathbf{X})(\mathbf{Y}-\mathbf{B}\mathbf{X})^T. & (1.5) \end{aligned}$$

Analogously, the quadratic form (1.2) implicitly defines the Hessian $\mathbf{H}$ through the identity

$$\begin{aligned} Q(\mathbf{U},\mathbf{V}) &= \operatorname{tr}\left\{ \begin{bmatrix} \operatorname{vec}(\mathbf{U})^T & \operatorname{vec}(\mathbf{V})^T \end{bmatrix} \begin{pmatrix} \mathbf{H}_{\mathbf{BB}} & \mathbf{H}_{\mathbf{B\Gamma}} \\ \mathbf{H}_{\mathbf{\Gamma B}} & \mathbf{H}_{\mathbf{\Gamma\Gamma}} \end{pmatrix} \begin{bmatrix} \operatorname{vec}(\mathbf{U}) \\ \operatorname{vec}(\mathbf{V}) \end{bmatrix} \right\} \\ &\equiv -\frac{n}{2}\operatorname{tr}\left[\mathbf{\Gamma}^{-1}\mathbf{V}\mathbf{\Gamma}^{-1}\mathbf{V}\right] + \frac{1}{2}\operatorname{tr}\left[(\mathbf{Y}-\mathbf{B}\mathbf{X})\mathbf{X}^T\mathbf{U}^T\mathbf{V}\right] \\ &\quad + \frac{1}{2}\operatorname{tr}\left[\mathbf{U}\mathbf{X}(\mathbf{Y}-\mathbf{B}\mathbf{X})^T\mathbf{V}\right] + \operatorname{tr}\left[\mathbf{X}(\mathbf{Y}-\mathbf{B}\mathbf{X})^T\mathbf{V}\mathbf{U}\right] - \operatorname{tr}(\mathbf{X}^T\mathbf{U}^T\mathbf{\Gamma}\mathbf{U}\mathbf{X}). \end{aligned}$$

Because $\mathrm{E}(\mathbf{Y}) = \mathbf{B}\mathbf{X}$, the expected information $\mathbf{J} = \mathrm{E}(-\mathbf{H})$ has the off-diagonal blocks $\mathbf{J}_{\mathbf{B},\mathbf{\Gamma}} = \mathbf{0}_{pr \times r^2}$ and $\mathbf{J}_{\mathbf{\Gamma},\mathbf{B}} = \mathbf{0}_{r^2 \times pr}$. Now the Kronecker product identity $\operatorname{vec}(\mathbf{ABC}) = (\mathbf{C}^T \otimes \mathbf{A}) \operatorname{vec}(\mathbf{B})$ implies

$$\begin{aligned} \operatorname{tr}(\mathbf{X}^T\mathbf{U}^T\mathbf{\Gamma}\mathbf{U}\mathbf{X}) &= \operatorname{tr}(\mathbf{X}\mathbf{X}^T\mathbf{U}^T\mathbf{\Gamma}\mathbf{U}) \\ &= \operatorname{tr}[(\mathbf{\Gamma}\mathbf{U}\mathbf{X}\mathbf{X}^T)^T\mathbf{U}] \\ &= \operatorname{vec}(\mathbf{\Gamma}\mathbf{U}\mathbf{X}\mathbf{X}^T)^T \operatorname{vec}(\mathbf{U}) \\ &= \left[(\mathbf{X}\mathbf{X}^T \otimes \mathbf{\Gamma}) \operatorname{vec}(\mathbf{U})\right]^T \operatorname{vec}(\mathbf{U}) \\ &= \operatorname{vec}(\mathbf{U})^T (\mathbf{X}\mathbf{X}^T \otimes \mathbf{\Gamma}) \operatorname{vec}(\mathbf{U}). \end{aligned}$$

It follows that $\mathbf{J}_{\mathbf{BB}} = \mathbf{X}\mathbf{X}^T \otimes \mathbf{\Gamma}$. Similarly,

$$\begin{aligned} \operatorname{tr}(\mathbf{\Gamma}^{-1}\mathbf{V}\mathbf{\Gamma}^{-1}\mathbf{V}) &= \left[(\mathbf{\Gamma}^{-1} \otimes \mathbf{\Gamma}^{-1}) \operatorname{vec}(\mathbf{V})\right]^T \operatorname{vec}(\mathbf{V}) \\ &= \operatorname{vec}(\mathbf{V})^T [\mathbf{\Gamma}^{-1} \otimes \mathbf{\Gamma}^{-1}] \operatorname{vec}(\mathbf{V}), \end{aligned}$$

so that $\mathbf{J}_{\boldsymbol{\Gamma\Gamma}} = \boldsymbol{\Gamma}^{-1} \otimes \boldsymbol{\Gamma}^{-1}$. In summary, the expected information matrix takes the block diagonal form

$$\mathbf{J} \;=\; \begin{pmatrix} (\mathbf{X}\mathbf{X}^T) \otimes \boldsymbol{\Gamma} & \mathbf{0} \\ \mathbf{0} & \boldsymbol{\Gamma}^{-1} \otimes \boldsymbol{\Gamma}^{-1} \end{pmatrix}. \tag{1.6}$$

In our projected steepest ascent algorithm, the expected information matrix is never explicitly formed. It is implicitly accessed in the step-size calculation through the associated quadratic form $Q(\mathbf{B}, \boldsymbol{\Gamma})$.

1.8 Full IHT Step Size

Let $(\mathbf{B}, \boldsymbol{\Gamma})$ be the matrix of regression coefficients and variance components obtained by horizontally contatentaing $\boldsymbol{\Gamma} \in \mathbb{R}^{r \times r}$ to $\mathbf{B} \in \mathbb{R}^{r \times p}$. The next iterate in full IHT is the projection of the point

$$\boldsymbol{\Delta}_{m+1} \;=\; (\mathbf{B}_m, \boldsymbol{\Gamma}_m) + t_m \nabla \mathcal{L}(\mathbf{B}_m, \boldsymbol{\Gamma}_m) \;=\; (\mathbf{B}_m, \boldsymbol{\Gamma}_m) + t_m (\mathbf{C}_m, \mathbf{W}_m), \tag{1.7}$$

where $\mathbf{C}_m = \nabla_{\mathbf{B}} \mathcal{L}$ and $\mathbf{W}_m = \nabla_{\boldsymbol{\Gamma}} \mathcal{L}$ evaluated at $(\mathbf{B}_m, \boldsymbol{\Gamma}_m)$. The loglikelihood along the ascent direction is a function of the scalar t_m and can be approximated by the second-order expansion

$$\mathcal{L}(\boldsymbol{\Delta}_{m+1}) \;\approx\; \mathcal{L}(\mathbf{B}_m, \boldsymbol{\Gamma}_m) + t_m \operatorname{tr}\left[(\mathbf{C}_m, \mathbf{W}_m)^T (\mathbf{C}_m, \mathbf{W}_m)\right] - \frac{t_m^2}{2} \operatorname{tr}\left[(\mathbf{C}_m, \mathbf{W}_m)^T \mathbf{J}(\mathbf{B}_m, \boldsymbol{\Gamma}_m)(\mathbf{C}_m, \mathbf{W}_m)\right].$$

The choice

$$t_m \;=\; \frac{\operatorname{tr}\left[(\mathbf{C}_m, \mathbf{W}_m)^T (\mathbf{C}_m, \mathbf{W}_m)\right]}{\operatorname{tr}\left[(\mathbf{C}_m, \mathbf{W}_m)^T J(\mathbf{B}_m, \boldsymbol{\Gamma}_m)(\mathbf{C}_m, \mathbf{W}_m)\right]} \;=\; \frac{\|(\mathbf{C}_m, \mathbf{W}_m)\|_F^2}{\operatorname{tr}(\mathbf{X}^T \mathbf{C}_m^T \boldsymbol{\Gamma}_m \mathbf{C}_m \mathbf{X}) + \frac{m}{2} \operatorname{tr}(\boldsymbol{\Gamma}_m^{-1} \mathbf{W}_m \boldsymbol{\Gamma}_m^{-1} \mathbf{W}_m)}$$

maximizes the approximation. If the support of the matrix $(\mathbf{B}, \boldsymbol{\Gamma})$ does not change under projection, then this IHT update is particularly apt.

1.9 IHT Projection

Recall that full IHT iterates according to

$$(\mathbf{B}_{m+1}, \boldsymbol{\Gamma}_{m+1}) \;=\; P_{S_k}(\boldsymbol{\Delta}_{m+1}),$$

where $\boldsymbol{\Delta}_{m+1}$ is derived in equation (1.7). Here k is a positive integer representing the sparsity level, which is assumed known. In practice k is found through cross-validation. The projection $P_{S_k}(\boldsymbol{\Delta})$ splits into separate projections for $\mathbf{B}$ and $\boldsymbol{\Gamma}$. One can independently project each row of $\mathbf{B}$ to sparsity. Alternatively, one can require each row of $\mathbf{B}$ to have the same sparsity pattern if the same set of predictors plausibly contribute to all r traits. The $\boldsymbol{\Gamma}$ projection must preserve symmetry and positive semidefiniteness. Symmetry is automatic because the gradient of $\boldsymbol{\Gamma}$ is already symmetric. To project to positive semidefiniteness, one takes the singular value decomposition of $\boldsymbol{\Gamma}$ and project its eigenvalues $\boldsymbol{\lambda}$ to nonnegativity. One can even project $\boldsymbol{\Gamma}$ to the closest positive definite matrix with an acceptable condition number [6].

1.10 The Block Ascent IHT

In block ascent we alternate updates of $\mathbf{B}$ and $\mathbf{\Gamma}$. The exact update (eq 4 of main text) of $\mathbf{\Gamma}$ is particularly convenient, and we take advantage of it. Symmetry and positive semidefiniteness are automatically preserved. Inversion can be carried out via Cholesky factorization of $\mathbf{\Gamma}$. This choice of $\mathbf{\Gamma}$ simplifies the step length

$$t_m = \frac{\|\mathbf{C}_m\|_F^2}{\text{tr}(\mathbf{X}^T\mathbf{C}_m^T\mathbf{\Gamma}\mathbf{C}_m\mathbf{X})}.$$

Note that the denominator of the step size does not require formation of the $n \times n$ matrix $\mathbf{X}^T\mathbf{C}_m^T\mathbf{\Gamma}\mathbf{C}_m\mathbf{X}$. One can write $\text{tr}(\mathbf{X}^T\mathbf{C}_m^T\mathbf{\Gamma}\mathbf{C}_m\mathbf{X}) = \text{tr}(\mathbf{X}^T\mathbf{C}_m^T\mathbf{L}\mathbf{L}^T\mathbf{C}_m\mathbf{X}) = \|\mathbf{L}^T\mathbf{C}_m\mathbf{X}\|_F^2$, where $\mathbf{L}$ is the Cholesky factor of $\mathbf{\Gamma}$. The matrix $\mathbf{L}^T\mathbf{C}_m\mathbf{X}$ is fortunately only $r \times n$.

1.11 UK Biobank Runtime Script

Here is the script used to perform our UK Biobank analysis

```
#
# Parameter explanations
# MvNormal: Distribution of traits is multivariate normal
# q: number of cross-validation folds
# min_iter: iterate at least 10 times before checking for convergence
#

using MendelIHT, Random, LinearAlgebra
BLAS.set_num_threads(1)
Random.seed!(2022)
plinkfile = "ukb.merged.metabolic.subset.european.400K.QC"
phenotypes = "traits.reordered.standardized.csv"
covariates = "covariates.reordered.standardized.csv"

# cross validate 1000, 2000, ..., 10000
path = 1000:1000:10000
@time mses = cross_validate(plinkfile, MvNormal, path=path, q=3,
    covariates=covariates, phenotypes=phenotypes, min_iter=10,
    cv_summaryfile="cviht.summary.roughpath1.txt")

# cross validate 3100, 3200, ..., 4900
k_rough_guess = path[argmin(mses)]
```

```
path = (k_rough_guess - 900):100:(k_rough_guess + 900)
@time mses = cross_validate(plinkfile, MvNormal, path=path, q=3,
    covariates=covariates, phenotypes=phenotypes, min_iter=10,
    cv_summaryfile="cviht.summary.roughpath2.txt")

# cross validate 4510, 4520, ..., 4690
k_rough_guess = path[argmin(mses)]
path = (k_rough_guess - 90):10:(k_rough_guess + 90)
@time mses = cross_validate(plinkfile, MvNormal, path=path, q=3,
    covariates=covariates, phenotypes=phenotypes, min_iter=10,
    cv_summaryfile="cviht.summary.roughpath3.txt")

# cross validate 4671, 4672, ..., 4689
k_rough_guess = path[argmin(mses)]
path = (k_rough_guess - 9):(k_rough_guess + 9)
@time mses = cross_validate(plinkfile, MvNormal, path=path, q=3,
    covariates=covariates, phenotypes=phenotypes, min_iter=10,
    cv_summaryfile="cviht.summary.final.txt")

# run full IHT on k = 4678
K = path[argmin(mses)]
@time iht_result = iht(plinkfile, K, MvNormal,
    summaryfile = "iht.final.summary.txt",
    betafile = "iht.final.beta.txt",
    covariancefile = "iht.final.cov.txt",
    covariates=covariates, phenotypes=phenotypes, max_iter=2000)
```

2 Acknowledgements

This work is partially supported by National Institutes of Health grants T32-HG02536 (BC), R01-HG006139 (BC, KL, HZ), R35 GM141798 (KL, JS, HZ), R01-HG009120 (JS), K01DK106116 (JJZ), and R21HL150374 (JJZ); National Science Foundation grants DMS-1264153 (JS), DMS-2054253 (HZ, JZ), and IIS-2205441 (HZ, JZ); and a National Research Foundation of Korea (NRF) grant 2020R1A6A3A03037675 (SK) from the Korean government. The UCLA Institute for Digital Research and Education's Research Technology Group supplied computational and storage services through its Hoffman2 Shared Cluster.

3 Author Contributions

KL conceived the project. BC, JS, and KL devised the methods and simulations. BC, SK, and HZ wrote the software. JZ and AJ accessed and analyzed the UK Biobank data. BC wrote the initial draft of the paper. All authors reviewed and edited the draft.

4 Competing interests

The authors declare no competing interests.

5 Web Resources

Project name: MendelIHT.jl
Project home page: `https://github.com/OpenMendel/MendelIHT.jl`
Supported operating systems: Mac OS, Linux, Windows
Programming language: Julia (unit tests pass on Julia 1.6 and 1.7 but MendelIHT.jl should work with all Julia 1.x versions)
License: MIT
All outputs and commands needed to reproduce the following results are available at the `MendelIHT` site in the `manuscript` sub-folder. `SnpArrays.jl` is available at `https://github.com/OpenMendel/SnpArrays.jl`. `VCFTools.jl` is available at `https://github.com/OpenMendel/VCFTools.jl`. `BGEN.jl` is available at `https://github.com/OpenMendel/BGEN.jl`

References

[1] M. C. Delfour. *Introduction to Optimization and Semidifferential Calculus*. SIAM, 2012.

[2] K. Lange. *MM Optimization Algorithms*, volume 147. SIAM, 2016.

[3] J. MacArthur, E. Bowler, M. Cerezo, L. Gil, P. Hall, E. Hastings, H. Junkins, A. McMahon, A. Milano, J. Morales, et al. The new NHGRI-EBI catalog of published genome-wide association studies (GWAS Catalog). *Nucleic Acids Research*, 45(D1):D896–D901, 2017.

[4] R. Magno and A.-T. Maia. gwasrapidd: an R package to query, download and wrangle GWAS Catalog data. *Bioinformatics*, 36(2):649–650, 2019.

[5] J. R. Magnus and H. Neudecker. *Matrix Differential Calculus with Applications in Statistics and Econometrics , 2nd edition*. John Wiley & Sons, 2019.

[6] M. Tanaka and K. Nakata. Positive definite matrix approximation with condition number constraint. *Optimization Letters*, 8(3):939–947, 2014.
